# Controlling Meiotic Recombinational Repair – Specifying the Roles of ZMMs, Sgs1 and Mus81/Mms4 in Crossover Formation

**DOI:** 10.1371/journal.pgen.1004690

**Published:** 2014-10-16

**Authors:** Ashwini Oke, Carol M. Anderson, Phoebe Yam, Jennifer C. Fung

**Affiliations:** Department of Obstetrics, Gynecology and Reproductive Sciences, Center of Reproductive Sciences, University of California, San Francisco, San Francisco, California, United States of America; National Cancer Institute, United States of America

## Abstract

Crossovers (COs) play a critical role in ensuring proper alignment and segregation of homologous chromosomes during meiosis. How the cell balances recombination between CO vs. noncrossover (NCO) outcomes is not completely understood. Further lacking is what constrains the extent of DNA repair such that multiple events do not arise from a single double-strand break (DSB). Here, by interpreting signatures that result from recombination genome-wide, we find that synaptonemal complex proteins promote crossing over in distinct ways. Our results suggest that Zip3 (RNF212) promotes biased cutting of the double Holliday-junction (dHJ) intermediate whereas surprisingly Msh4 does not. Moreover, detailed examination of conversion tracts in *sgs1* and *mms4-md* mutants reveal distinct aberrant recombination events involving multiple chromatid invasions. In *sgs1* mutants, these multiple invasions are generally multichromatid involving 3–4 chromatids; in *mms4-md* mutants the multiple invasions preferentially resolve into one or two chromatids. Our analysis suggests that Mus81/Mms4 (Eme1), rather than just being a minor resolvase for COs is crucial for both COs and NCOs in preventing chromosome entanglements by removing 3′- flaps to promote second-end capture. Together our results force a reevaluation of how key recombination enzymes collaborate to specify the outcome of meiotic DNA repair.

## Author Summary

A critical component of successful reproduction is ensuring that the correct number of chromosomes is distributed to the gametes (i.e. sperm, eggs). Incorrect numbers of chromosomes in our gametes can directly result in infertility, miscarriages and developmental disabilities such as Down syndrome. Gamete production involves meiosis, in which crossovers between parental chromosomes are required to promote proper chromosome segregation. However, other types of recombination can occur that are not productive towards appropriate chromosome segregation. In this study, we examine several genes that are thought to play important roles in crossover (CO) promotion. By interpreting the final recombination products using a sequencing based analysis of all four gametes of an individual meiosis in budding yeast, we can infer the roles of these genes in recombination. We find that one protein, Zip3, can direct biased cleavage of the dHJ intermediate but another protein, Msh4, in the same complex cannot. Moreover, we find that a minor resolvase, Mus81/Mms4 (Eme1) is crucial in limiting chromosome entanglements by suppressing multiple consecutive recombination events from initiating from a single double-strand break (DSB). We favor a model that Mms4 is needed to remove a 3′-flap such that second-end capture of the DSB can occur.

## Introduction

Homologous recombination during meiosis plays an integral role in ensuring that each gamete receives exactly one copy of each chromosome from its diploid parent. COs, representing reciprocal repair between homologs, become chiasmata – physical bridges between homologous chromosomes that are required for the proper alignment and subsequent segregation of the homologs during the first meiotic division. Perturbation of crossing over leads to missegregation of chromosomes resulting in infertility, developmental disabilities and miscarriages [1]. Given the adverse consequences stemming from problems in crossing over, there is a clear need to understand the underlying mechanisms by which COs are controlled, particularly how the cell balances the choice of partner for recombination: intersister (IS) vs. interhomolog (IH) and the choice in pathway: reciprocal exchange resulting in COs vs. nonreciprocal exchange resulting in NCOs.

**Figure 1:**
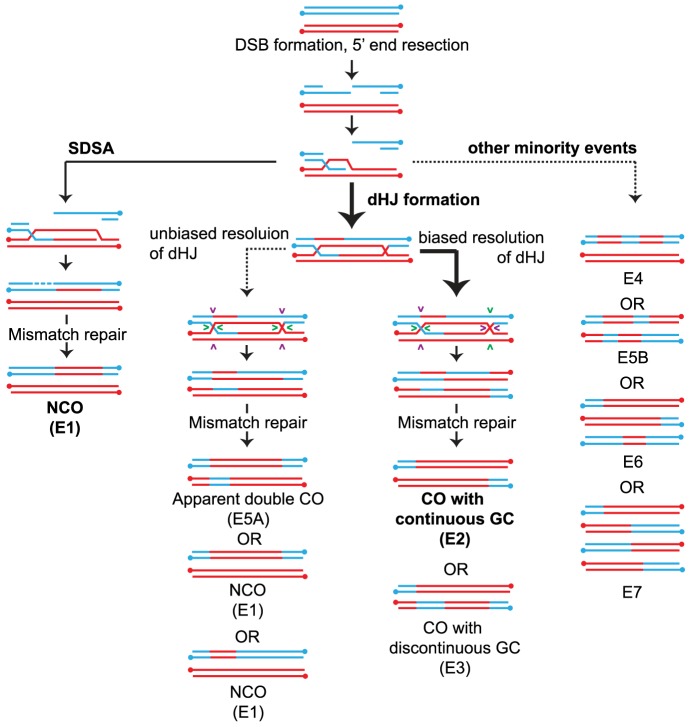
Overview of meiotic recombination pathways. COs and NCOs normally form through two different pathways involving invasion of one of the resected 3′ ends of the DSB into a homolog followed by DNA repair (a) NCOs arise via SDSA where 3′ strand invasion is not accompanied by formation of a stable JM between the homologs. Disassembly of the SDSA intermediate allows reannealing and ligation to the other end of the DSB resulting in a NCO. (b) dHJ formation where the strand invasion is accompanied by stable JM formation. The majority of the dHJs are resolved into COs by biased cuts made to the two HJs followed by mismatch repair of the heteroduplexes. (Details about how heteroduplexes are resolved are shown in Figure S1). Pairs of cuts are indicated by similar colored arrowheads. A small fraction of the dHJs can resolve to form NCOs by unbiased cutting of the two HJs. (c) In addition, a minority of events likely results from multistrand invasions. Event classifications (E#) are described in the legend of Figure 2. Arrow weight indicates the degree of partitioning through the various pathways in WT.

Based on budding yeast studies [2]–[4], COs are thought to mainly arise from biased resolution of dHJ intermediates that can be observed physically as joint molecules (JM) using 2D gels [5]. This is not the case for NCOs. Although a minority of NCOs may arise through unbiased cutting of the JM [6] (Figure 1), the bulk of NCOs appears to form via synthesis-dependent strand annealing (SDSA) [7]–[9] or by topoisomerase-assisted dissolution [8]. NCO formation is temporally distinct from CO formation, since NCOs appear about 30 minutes earlier than JM resolution [2], [10]. The difference in the formation of COs and NCOs is further highlighted by the fact that NCO formation is independent of Cdc5, a polo-like kinase, whereas COs require Cdc5 activity for JM resolution [11]. Taken together these studies clearly point to distinct mechanisms and intermediates that exist in the formation of COs vs. NCOs during meiosis.

Recently, Sgs1, the yeast analog to the RecQ family helicase BLM has been identified as having a major role in directing recombinational repair in meiosis into either a NCO or JM fate [2], [12], [13]. Sgs1/BLM is a 3′-5′ helicase that is characterized as being part of an anti-CO complex or “dissolvasome” that can take apart DNA structures that stem from DNA replication or homologous recombination [14], [15]. In vitro, BLM can disrupt D-loops [16] and can displace a Rad51-coated single stranded DNA filament [17]. During meiosis, this type of displacement is postulated to lead to NCO formation by SDSA. The ability of Sgs1/BLM to branch migrate dHJs together followed by decatenation of TopoIIIA mediated single strand exchange (reviewed in [18]) is another mode by which meiotic NCOs can form, thereby diverting events away from a fate that would otherwise lead to COs. This ability of Sgs1/BLM to dismantle DNA structures has been taken to suggest that Sgs1 is also needed to disassemble aberrant recombination intermediates, an idea that is supported by the elevated levels of multichromatid recombination intermediates involving three or four chromatids in an *sgs1* mutant [19].

In meiotic cells, Sgs1 exhibits an antagonistic relationship to synaptonemal complex proteins [19], [20] whose ability to promote crossing overs counters Sgs1 anti-CO activity [21]. The synaptonemal complex is a structure that resides between homologous chromosomes and is responsible for chromosome synapsis and CO promotion during meiotic prophase I. The synaptonemal complex proteins, collectively called ZMMs (Zip1/2/3/4(Spo22) Msh4/5 and Mer3), contribute in various ways to initiate synapsis and/or promote crossing over [22]. *zmm* mutants result in significant reductions in synapsis and crossing over. In *ZMM* deletion strains, removing Sgs1 and thus the ability to strand displace or promote dissolution of the D-loop restores crossing over to near wild-type levels [20]. ZMMs might protect JMs in at least two possible ways: 1) by providing an environment in which Sgs1 is unable to dismantle the JM intermediate and/or 2) by setting up a configuration in which biased cutting of the JM directs resolution solely towards the CO outcome.

Besides Sgs1, other recombination enzymes have been intensively investigated for their roles in meiotic CO formation. Recently, Mus81/Mms4 (Eme1), Slx1–Slx4, Yen1 and MutLγ-Exo1 have been shown to account for essentially all JM resolution in budding yeast, with the majority of meiotic JM resolution (∼49%) originating from the MutLγ-Exo1 pathway [13]. Although playing a major role in JM resolution in fission yeast [23], [24], Mus81/Mms4 (Eme1) is thought to have a reduced role in budding yeast [13]. Interestingly, both the sporulation efficiency (<12%) and spore viability (<51%) of a *mms4* null mutant are more severely defective [25], [26] than the sporulation efficiency (73%) and spore viability (79%) of a MutLγ mutant such as *mlh3* [27]. This might suggest that Mlh3 cannot resolve Mms4-dependent JMs or that there might be additional functions for Mus81/Mms4 (Eme1) beyond its JM resolution capabilities. Mus81-Mms4 (Eme1) has homology to XPF family endonucleases that are involved in nucleotide excision repair [28]. In vitro studies have shown that Mus81-Mms4 (Eme1) is efficient in cutting branched structures such as 3′ overhangs, forks, nicked HJs and D-loops [29]. It has been proposed that Mus81/Mms4 (Eme1) is needed to cleave 3′ flaps, created by overextension of DNA synthesis in the D-loop. This cleavage would promote ligation to the 5′ end permitting second end capture [25], [30]. In accordance, *mms4* null mutants show larger gene conversion tracts [30]. However, based on biochemical characterizations of recombinant Mus81 and TEV-Mus81 cleavage activity [31], [32], this model fell into obscurity in lieu of the idea that Mus81/Eme1(Mms4) instead cleaves D-loops and half-junctions in order to resolve dHJs [33].

Recently, the development of high density oligonucleotide microarray mapping and high throughput sequencing of single nucleotide polymorphisms (SNPs) in meiotic tetrads has allowed detailed examination of the genome-wide distribution and composition of resolution patterns for both NCOs and COs in a method we now term RecSeq [6], [34]–[36]. In a yeast hybrid strain, a cross between YJM789 and S96, ∼60,000 SNPs can be genotyped to assess parental origin of DNA in progeny from the two strains [37]. Because each of the four products of a single meiosis can be isolated from a tetrad, NCOs and COs can be clearly distinguished by SNP analysis.

Here, we perform an extensive analysis of tetrads from *sgs1*, *zip3*, *msh4*, and *mms4-md* strains to further delineate the process by which COs and NCOs are formed. Through analysis of changes in the GC tract composition within COs and NCOs, we identify a recombination signature indicative of unbiased cleavage of dHJ intermediates for *sgs1* and *zip3*, but not for *msh4*. In addition, detailed examination of conversion tracts in *sgs1* and *mms4-md* mutants reveal distinct aberrant recombination events involving multiple chromatid invasions. In *sgs1* mutants, these multiple invasions are generally multichromatid involving 3–4 chromatids; in *mms4-md* mutants the multiple invasions preferentially resolve into one or two chromatids. We suggest that Mms4 is needed to prepare the invading strand to properly dock with the second end of the double strand break (DSB) thus ensuring only a single round of invasion for both NCOs and COs. The loss of this alternative role for Mus81/Mms4, rather than removal of its function as a resolvase, could contribute to extreme loss of viability seen in gametes that lack Mus81/Mms4.

## Results

### Genome-Wide Analysis of Recombination in *sgs1*, *zip3* and *msh4* Mutants

It has been proposed that Sgs1 determines the outcome of NCOs and COs through an antagonistic relationship between itself and ZMM proteins [20]. To better understand this relationship between Sgs1 and the ZMM proteins, we turned to high throughput sequencing to ascertain the number, distribution and composition of COs and NCOs genome-wide in eleven *sgs1*, seven *zip3*, seven *msh4*, four *zip3 sgs1* and five *msh4 sgs1* tetrads that we compared to 52 wild-type tetrads. The wild-type tetrads combine six tetrads from sequencing [38] and 46 tetrads from high-density oligo microarrays [35] that we reanalyzed using our new classification scheme described below.

**Figure 2:**
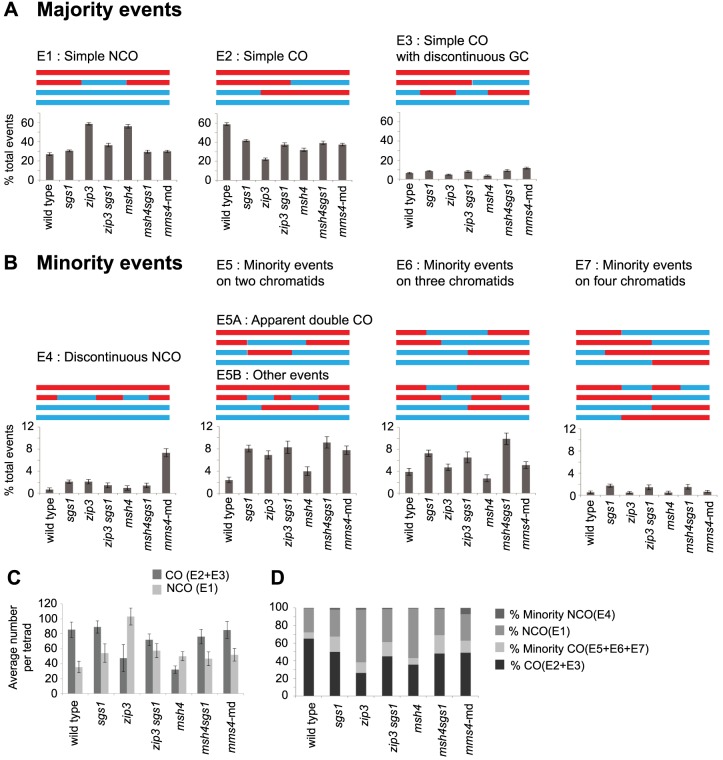
Types of recombination signatures expected and observed by RecSeq. (A) The majority events are recombination signatures expected from normal SDSA and biased cleavage of JMs. Percentage of these events found for the various mutants are shown. Total events = E1–E7. (B) The minority events are recombination signatures that are found more rarely. Percentage of these events found for the various mutants are shown and can also be found in Table S1. WT has very few of these events. E1: Simple NCO is a 3∶1 tract on one chromatid and is not within 5 kb of another NCO or CO. E2: Simple CO is a CO with or without associated GC tracts and not within 5 kb of another CO or NCO. E3: Simple CO with discontinuous GC is a CO with or without associated GC tract with one or more GC within 5 kb and on one of the same chromatids as the CO chromatids. E4: Discontinuous NCO is two or more NCOs in a tandem fashion on one chromatid with regions of 2∶2 marker segregation separating them. E5 is an event where two or more COs or NCOs are within 5 kb of each other and involve only two chromatids. E5 is subdivided into E5A and E5B where E5A represents the signature of unbiased dHJ resolution and E5B represents all other E5 events. E6 is an event where two or more COs or NCOs are within 5 kb of each other and involve three chromatids. E7 is an event where two or more COs or NCOs are within 5 kb of each other and involve four chromatids. (Error bars – SE of proportions). T-test statistics comparing each mutant to another and WT (Table S2). (C) Average number of COs and NCOs per tetrad. Error bars, SD (D) Proportion of COs, NCOs and minority events out of total events.

We first sorted recombination events into majority and minority categories (Figure 2) then subdivided events based on the number of chromatids involved. For wild type (WT), the majority of DSBs are repaired as either a NCO (Figure 2A, E1) or CO (Figure 2A, E2 and E3). Together these form the “majority” events since they are the dominant form of IH recombination events (92.4%) and the predicted outcomes of SDSA and biased dHJ resolution (Figure 1, Figure S1). Note that NCOs can also form from unbiased dHJ dissolution and unbiased dHJ resolution (Figure S1), though this is normally suppressed in wild-type strains. We named the remaining IH events that are seen less frequently “minority” events that include cases where two or more COs, NCOs or CO-NCOs are within 5 kb of each other (Figure 2B, E4–7). Most of these recombination events cannot be explained by simple resolution of a dHJ as shown in Figure 1 and therefore must arise from atypical resolution involving either multiple invasions or multiple nearby DSBs. Minority events make up 7.6% of all WT events (Figure 2B) and can potentially arise from a single DSB as demonstrated in Figure S2A. Of the total events, 4.4% are multichromatid (events on three or four chromatids) averaging 5.8 per meiosis (Figure 2B, E6–7). This finding is consistent with Oh et al. (2007) showing that probable intermediates of these events do occur in wild-type cells but in low abundance.

Note that some minority events, particularly E5A events can potentially arise through unbiased cutting of the dHJ (Figure 1, Figure S1). E5A events could also arise from two independent DSBs that get repaired as two NCOs. There are three possible configurations that could occur if two DSBs are very close to each other: (A) overlapping NCOs on a pair of homologous chromosomes, (B) non- overlapping NCOs on a pair of homologous chromosomes or (C) NCOs on sister chromatids. If these double NCOs arise from independent DSBs, we would expect each of these configurations to be equally likely. However, overlapping NCOs (E5A) form the majority of all E5 events (83 out of 167) (Figure S3) suggesting that these are more likely to arise from unbiased resolution of dHJ.

### Distinct Signatures Indicative of Unbiased Resolution of dHJs Occur in *sgs1 and zip3*

In the presence of Sgs1 and ZMMs, JMs are thought to resolve predominantly by Exo1-MutLγ that cleaves the dHJ in a biased direction to direct resolution of the dHJs to form COs [39]. However in the absence of Sgs1, JMs become dependent on Mus81/Mms4, Slx1-Slx4 and Yen1 for resolution [13]. Dependence on these resolvases results in the loss of CO bias presumably because of unbiased cutting of the JM. Similarly, ZMMs are thought to play a role in ensuring biased resolution of the dHJ. But do all ZMMs contribute in the same way?

To determine whether unbiased cleavage is indeed occurring in the *sgs1* and *zmm* mutants, we examined whether we could detect an increase in apparent double COs or E5A events per meiosis. E5A events are distinct recombination signatures predicted to arise if the cuts to the JMs are no longer biased (Figure 1, Figure S1). In agreement with the occurrence of unbiased cleavage in *sgs1*, we do observe an increase in number of E5A events per tetrad (5.7 *sgs1* vs. 1.6 WT p = 0.0003, Table S1). The *zip3* mutant also exhibits an increase in E5A events (3.6 *zip3* vs. 1.6 WT p = 0.0122, Table S1). In contrast, *msh4* shows a decrease in the number of E5A events (0.6 *msh4* vs. 1.6 WT p = 0.0003) proportional to a decrease in CO levels. From these results, we infer that both Sgs1 and Zip3 normally prevent unbiased cutting of JMs, however Msh4 does not. In *zip3*, more NCOs arise either from SDSA and/or from unbiased dHJ cleavage likely contributing to the dramatic increase in NCOs in this mutant (Figure 2C). Correspondingly in *msh4*, the wild-type level of NCOs (Figure 2C) and the lack of unbiased signatures for this mutant are in accordance with NCOs arising predominantly from normal levels of SDSA.

### Proportion of CO to NCO Levels Changes Dramatically in *zmm* Mutants but Not in *sgs1*

To investigate whether Sgs1 affects the CO-NCO decision via dictating the relative amounts of COs and NCOs, we examined the average CO and NCO levels in the *sgs1* mutant. We find that overall levels of NCOs increase (t-test p value = <0.001) while CO levels of the majority class (E2,E3) remain unchanged (t-test p value = 0.21) (Figure 2C, Table S1). It follows that there is a small but significant decrease in the relative proportion of COs of the majority class (50%) to NCOs (31%) as compared to WT (z test of proportions p value = <0.001), for which 65% of the observed IH events are COs and 27% are NCOs (Figure 2D). In the case of the two null mutants of the ZMM genes *ZIP3* and *MSH4* a more dramatic change is observed. The relative proportion of COs to NCOs in both *zip3* and *msh4* deviate substantially from that of WT (*zip3-* 39% CO, 60.9% NCO; msh4- 43% CO, 57% NCO, z test of proportions p value = <0.0001 for both) (Figure 2D). Removing Sgs1 in conjunction with eliminating either *ZMM* restores the proportion of COs and NCOs to near *sgs1* levels [20]. Thus it seems that *zmm* mutants do affect the CO-NCO outcome through substantial changes to the CO/NCO ratio, but only when Sgs1 is present. Unlike the ZMMs, Sgs1's role in dictating the CO-NCO decision does not occur through major changes in the ratio of COs to NCOs, whether ZMMs are present or not.

### Zip3-Dependent Population of Short NCOs Becomes Evident in the *sgs1* Mutant

**Figure 3:**
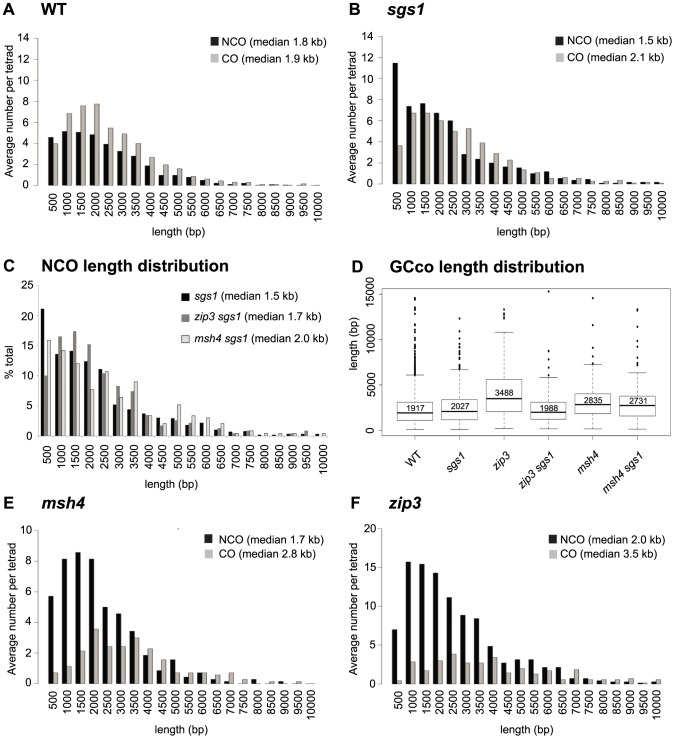
Distribution of GCco and NCO tract lengths for *sgs1* and *zmm* mutants. (A) Comparison of GCco vs. NCO tract lengths for WT (B) Comparison of GCco vs. NCO tract lengths for *sgs1*. (C) NCO length distributions for *sgs1*, *msh4sgs1* and *zip3sgs1* (D) Box plot of GC_CO_ lengths. The dark line in each box represents median GC_CO_ length. Each box outlines lower and upper quartile. The whiskers denote the boundaries of the interquartile range and the circles outside the whiskers represent outliers (outside 1.5 times the interquartile range). The value inside each box is the median GC_CO_ length. (E) Comparison of GC_CO_ vs. NCO tract lengths for *msh4* (F) Comparison of GC_CO_ vs. NCO tract lengths for *zip3*.

The distributions of NCO and CO-associated GCs (GC_CO_s) tract lengths are different for WT, both for the medians (Wilcoxon rank sum p<0.0001) [35] and for the shapes of the distribution (Kolmogorov-Smirnov (K-S), p<0.001) (Figure 3A). The distinctive distributions are not surprising given that COs and NCOs normally arise from separate intermediates. In *sgs1*, it was proposed that COs and NCOs arise from the same JM intermediate [10], [13] which led to our initial but incorrect prediction, that in this mutant, NCO and CO length distributions should closely overlap. Instead, we find that the two distributions do overlap for the most part except for one distinct difference. Notably, the NCO tract distribution is characterized by a novel population of short NCO tracts in the range of 0–500 bp in length (Figure 3B). These NCOs seem not to arise by a change in the restoration vs. conversion ratio since the we do not see a concomitant increase in the percentage of gene conversions associated with COs. Interestingly, the population of short NCOs seems dependent on Zip3, but not Msh4, since the *sgs1 zip3* double mutant lacks this cohort of short NCOs, but in *sgs1 msh4*, they are still present (Figure 3C – first bin). Given that NCO timing parallels CO timing in an *sgs1* mutant [2], [10], this suggests that the short NCOs may be a consequence of some NCOs forming from JMs or JM-like intermediates.

### *zip3* but Not *msh4* Exhibits Sgs1-Dependent Increases in GC_CO_ Tract Lengths

Lack of ZMMs might be predicted to result in increased tract lengths of GC_CO_s since ZMMs may oppose Sgs1's postulated role in D-loop extension. Indeed we find that the *zip3* mutant shows an increase in median GCco tract lengths as compared to WT (Figure 3D, 3F, Table S3 (p<0.001)). As expected, this increase is Sgs1-dependent, since GC_CO_ tracts exhibit wild-type lengths in *zip3 sgs1* (Figure 3D, Table S3 (p = 0.36)). Thus when Sgs1 is present, Zip3 is required to maintain wild-type lengths. This is consistent with the idea that Zip3 limits D-loop extension driven by Sgs1, by promoting efficient ligation at the second end of the DSB. Other evidence consistent with a role for Zip3 in promoting ligation is the increased frequency of minority events in *zip3* (Figure 2B, Table S1), since multiple invasions would be a likely consequence of inefficiently ligated 3′ ends. Another possibility is that Zip3 limits extensive branch migration; however, extensive branch migration has yet to be shown in wild-type yeast. For *msh4*, we also find that median GC_CO_ tract lengths are longer compared to WT, as was previously shown [35] (Figure 3D–3E Table S3 (p = <0.001)), though this increase was less than what was seen for *zip3*. Interestingly, the increase in tract length is independent of Sgs1 since in *msh4 sgs1*, the median GC_CO_ tract size is equivalent to *msh4* alone (Figure 3D, Table S3 (p = 0.23)). These results suggest that although Zip3 is needed to limit Sgs1 D-loop extension, Msh4 does not seem to be required. This is consistent with the observation that in a *msh4* mutant, Zip3 still localizes normally [40] and potentially still functions to limit extension at the ligating end. In the discussion, we speculate how the GC_CO_ tract lengths can increase in a *msh4* mutant without being dependent on Sgs1.

### *zip3* but Not *msh4* Exhibits an Increase in NCO Tract Lengths

We next examined NCO lengths. If NCOs predominantly occur via SDSA, we would not expect any change in NCO lengths in the *zmm* mutants since ZMM proteins are thought to be JM-specific. However, if NCOs are now being created through unbiased resolution of dHJs, we might expect a population of longer NCOs added to the normal population of NCOs occurring through SDSA. This is true for *zip3*. Here we see that NCO lengths increase by 184 bp, which is significantly longer than in WT (1960 bp *zip3*, 1778 WT, Wilcoxon p<0.0001, Table S1, Table S3). Although this only represents a ∼10% increase in median length, it is what might be expected given that the majority of NCOs likely still occur through SDSA. In contrast, in *msh4*, we observe no difference in the length of NCOs (1679 bp *msh4*, 1778 bp WT, Wilcoxon p = 0.19, Table S1, Table S3). Both the lack of increase in NCO conversion lengths and in E5As for this mutant suggests that NCOs do not arise through unbiased cutting in this *msh4*. Such findings further bolster the notion that Zip3 but not Msh4 influences biased cutting of the dHJ.

### *sgs1* Mutants Exhibit an Increased Number of Minority Events

The existence of multichromatid intermediates seen by physical analysis of recombination on 2D gels [19] predicts that an increase of such events should be observed in the final recombination products in *sgs1* mutants. To examine whether such events are indeed resolved in viable spores, we further examined the minority events for *sgs1* (Figure S2B). At the same time we wanted to analyze the *mms4-md* mutant since previous studies examining recombination intermediates suggest that Mus81/Mms4 (Eme1) collaborates with Sgs1 in resolving aberrant joint molecules [41], [42]. We find that minority events make up 19.2% of the total events for *sgs1* (p<0.0001) and 20.9% for *mms4-md* (p<0.0001) compared to only 7.6% in WT (Figure 2B E4–E7). This increase is not a general feature of all meiotic recombination mutants since *msh4* mutants show no significant change in the number of minority events (Figure 2B, E2, E4–E7). Further characterization of the minority events in *sgs1* reveals a significant increase in E5As as compared to WT (Figure S2B) as well as higher levels of more complex events involving multiple combinations of COs, NCOs and GCs engaging 2, 3 and 4 chromatids (Figure 2B E5–E7, Figure S2C). Multichromatid events are also significantly higher in *mms4-md* than in WT, but they are fewer in comparison to *sgs1*.

Minority events can potentially arise from a single DSB or multiple DSBs (Figure S2A). In *sgs1*, we have more IH events than WT (Table S1); this either means that there are fewer intersister events and/or potentially more DSBs. Since Oh et al. (2007) showed that there is more intersister recombination in an *sgs1* mutant, this raises the question whether minority events in *sgs1* are in fact independent COs repaired from more than one DSB. Sister chromatid ratios allow us to distinguish between these possibilities. If the closely spaced COs with a minority event consist of more than one independent event, we would expect a 1∶2∶1 ratio for apparent double COs on two, three and four chromatids respectively since normally COs do not demonstrate chromatid interference [43]. In *sgs1*, the observed ratio is 6∶7∶1 suggesting that the apparent closely spaced double COs making up a minority event likely stem from a single event.

### *mms4-md* Causes Discontinuities in NCO and GC_CO_ Tracts

In *mms4-md* tetrads, we find that multichromatid events increase compared to WT but to a lesser extent than observed in *sgs1* (Figure 2B: E5,E6,E7). Notably, *mms4-md* mutants preferentially exhibit prominent increases in events involving a discontinuous NCO (Figure 2B, E4) and in events in which COs exhibit discontinuous GCs (Figure 2A, E3). Therefore, although both *sgs1* and *mms4-md* demonstrate a similar change in the number of minority events, clear differences exist in the resolution signatures between *sgs1* and *mms4-md*.

**Figure 4:**
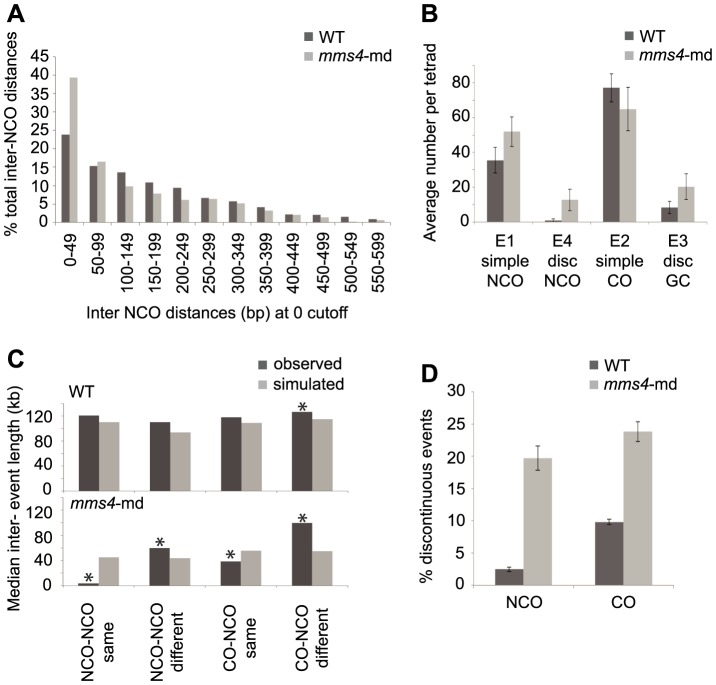
*mms4-md* results in an increase in discontinuous CO and NCO events. (A) Histogram of interNCO distances showing an increased frequency of closely spaced NCOs (1^st^ bin) in *mms4-md* vs. WT. NCOs were identified with a 0 kb cutoff and then interNCO distances were calculated between each pair of adjacent NCOs. (B) Average number of simple NCO, discontinuous NCO, CO with GCco and CO with discGCco per tetrad plotted for WT and *mms4-md*. (Error bars – SD). (C) A random distribution of NCOs across the genome was simulated using the observed number of NCOs in WT and *mms4-md* tetrads respectively. CO positions were left unchanged. Distances were calculated between adjacent NCOs on same chromatid (NCO-NCO same), adjacent NCOs on different chromatids (NCO-NCO different), adjacent CO and NCO on same chromatid (CO-NCO same) and adjacent CO-NCO on different chromatids (CO-NCO different). Median distance for each category was plotted for WT and *mms4-md*. Asterisks indicate statistical significance (D) Percent of total NCOs and COs that show discontinuity in WT and *mms4-md*. (Error bars - SE of proportions).

The observation that there are more discontinuities that are part of the same event in *mms4-md* arose from the analysis of NCOs. Initially, we observed an unexpectedly high number of NCOs (∼118 per tetrad as compared to 39 per WT tetrad). Intriguingly, most of the NCOs appeared to be closely spaced (Figure 4A). Although previous studies merged events by imposing a cutoff for CO-CO and CO-NCO distances, no distance cutoffs for NCO-NCO distances were typically used to determine whether two closely spaced NCOs are part of the same event. By imposing the same 5 kb cutoff as we used for CO-CO and CO-NCO, the number of NCOs was reduced to an average of 51.9 per tetrad rather than the 118 identified initially (Figure 4B). Furthermore, the discontinuities observed in *mms4-md* now consist of many more tracts than observed for WT, with a range of 2–11 sequential tracts seen for *mms4-md* compared to the typical 2–3 tracts for WT.

To rule out the possibility that the sheer number of NCOs when there is no cutoff would generate apparent discontinuities by random chance, we simulated over each tetrad's CO map a random distribution of NCOs using the experimentally determined NCO number from each tetrad. Calculating the distances between adjacent NCOs and between each CO and the adjacent NCO for the same and different chromatids, we then compared the simulated distances to the experimentally observed distances (Figure 4C). In WT, the observed distances between adjacent events for the majority of the cases do not differ greatly from the simulated values. Only for distances between CO-NCO on different chromatids do we see a small but significant difference between simulated and observed (Figure 4C). We thus can conclude for WT that the distribution of NCOs is generally, though not perfectly, consistent with a random dispersal of NCOs. This is in agreement with Mancera et al. (2008) who showed that NCOs and COs interfere with each other.

This is not the case for *mms4-md*. In *mms4-md*, all distances between observed and simulated distributions are different. Particularly striking is that distances between adjacent events on the same chromatid are significantly shorter than expected if NCOs were randomly distributed (Figure 4C). As a corollary, events on different chromatids are spaced farther apart than expected. Thus, in *mms4-md*, there is a strong chromatid bias for placement of adjacent NCOs such that they tend to be on the same chromatid. This argues that these closely spaced discontinuous NCOs are likely to arise from a single DSB. The same is also true for CO with discontinuous GC (discGC_CO_s) (Figure 4C). Surprisingly, a similar fraction of COs and NCOs show discontinuity in *mms4-md* (Figure 4D). On average we see 20.3 discGC_CO_s and 12.7 discontinuous NCOs per tetrad (Figure 4B, p value <0.05, p value<0.005, Table S1, Table S2) as compared to 8.4 and 0.9 in WT (Figure 4B, Table S1, Table S2). Since the absence of Mms4 affects NCO and GC formation to similar degrees, it suggests that Mms4 might act in a key mechanistic step common to the formation of both COs and NCOs.

### *mms4-md* Does Not Disrupt the Mismatch Repair Pathway

**Figure 5:**
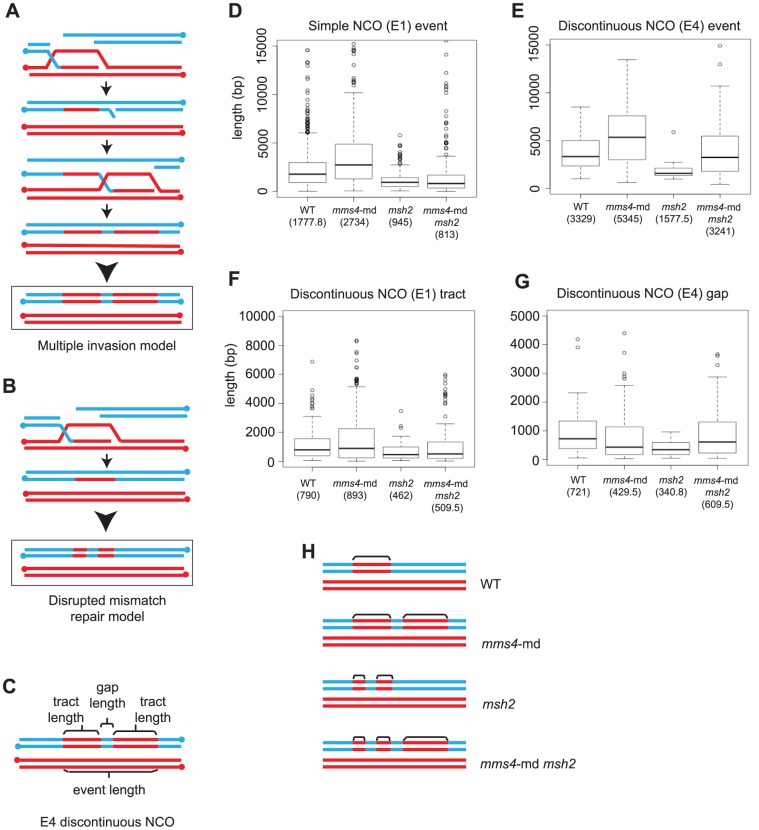
Multiple strand invasions can account for discontinuities seen in *mms4-md*. (A) Multiple strand invasions or (B) a defect in mismatch repair could potentially account for appearance of discontinuities in *mms4-md*. (C) Depiction of the various components of a discontinuous NCO event. (D) Box plot for tract lengths of simple NCOs (E1). The dark line in each box represents median length. Each box outlines lower and upper quartile. The whiskers denote minimum and maximum data and the circles outside the whiskers represent outliers (outside 1.5 times the inter quartile range). Values for the median length are given. (E) Box plot of event length for the entire discontinuous NCO. (F) Box plot of individual tract lengths within the discontinuous NCO. (G) Box plot of gap lengths between discontinuous NCOs. (H) Representation of observed discontinuous NCO events in *mms4-md*, *msh2* and *mms4-md msh2*. Note that discontinuous events only make up 24% of all NCOs in a *mms4-md* mutant.

Two possible models can explain the formation of discontinuous NCOs and GCs. One possibility is that without Mms4 each individual tract within the discontinuous event represents an IH invasion and repair cycle arising from multiple invasions (Figure 5A). The other possibility is that the mismatch repair pathway is disrupted by lack of Mms4 and the regions of heteroduplex DNA are not repaired completely giving rise to discontinuities (Figure 5B). In the disrupted mismatch repair model, NCO formation occurs normally, forming a heteroduplex. If the mismatch repair system is perturbed, this heteroduplex cannot be repaired completely. Partial mismatch repair within the heteroduplex DNA will create discontinuities.

Individual components of an entire discontinuous event can be measured (Figure 5C). The model in which mismatch repair is disrupted predicts discontinuous events in which the length of the entire event is similar to length of wild-type NCO and GC_CO_ tracts but individual conversion tracts within these events are shorter. In contrast for the multiple invasion model, because each invasion is an independent D-loop, the entire discontinuous event would be much longer while the individual conversion tracts would be similar in length to wild-type simple NCOs. We find that lengths of simple NCOs in WT are significantly shorter than lengths of discontinuous events in mms4-md (median length of 5.4 kb, Fig. 5D, 5E, Table S1). Importantly, individual tract of a discontinuous event in *mms4* are not different from the median tract length of a simple WT NCO (Figure 5D, 5F). Both of these observations are consistent with the view that in the absence of Mms4, discontinuous events arise through a multiple invasion pathway.

### In *mms4-md*, Discontinuous Events Likely Arise through Multiple Invasions

Further evidence that the multiple invasion model may explain the discontinuous events in *mms4-md* comes from examining the discontinuities that arise when mismatch repair is compromised by the elimination of Msh2. Msh2 recognizes and repairs heteroduplex DNA during recombination. In the absence of Msh2, heteroduplex DNA remains unrepaired and after the first division following meiosis, a mixed population of genotypes results. However, other Msh2-independent repair mechanisms such as the short patch repair system still function [44]. Because this short patch repair is not as efficient as Msh2 mismatch repair, we expect the GC tracts to be repaired less efficiently thus creating shorter tract lengths and discontinuities. In this case, we expect discontinuous events in which 2∶2 regions within the GC tract would represent restored/repaired regions.

In absence of Msh2, 10% of all the NCOs show discontinuity. The median length of an entire discontinuous NCO event in *msh2* is 1.6 kb, which is similar to the median length of a simple NCO in WT of 1.8 kb (p = 0.14) (Figure 5D, Figure 5E). The median length of conversion tracts within the discontinuous events is considerably shorter (0.5 kb) (Figure 5F) and the 2∶2 gap lengths between tracts are even shorter (0.3 kb) (Figure 5G) (Table S1). It is clear that the length distributions and discontinuity profiles of *mms4-md* and *msh2* are distinct. *mms4-md* mutants show very long discontinuous tracts and each tract within the discontinuity is similar in length to a simple NCO, whereas in *msh2*, the entire length of a discontinuous event is similar in size to the length of a simple NCO and the lengths of tracts within are shorter. Further bolstering the argument that disrupted mismatch repair is not causing the discontinuities in *mms4-md* is the observation that in an *msh2 mms4-md* double mutant the resulting discontinuous events appear to be a convolution of the independent phenotypes of *msh2* and *mms4-md* (Figure 5D–G). In Figure 5E, we see that in *msh2 mms4-md*, the entire discontinuous region is still long but the tracts making up the discontinuities are now short as in *msh2* (Figure 5F). Figure 5H summarizes our findings for the various mutants. Note that discontinuous events are ∼20% of the total NCOs and do not correspond to 100% of the events. We thus conclude that compromised mismatch repair does not create the discontinuities seen in the *mms4-md* mutant and that multiple strand invasions occurring for both NCOs and COs are the likely reason for the increased tandem tracts.

## Discussion

### RecSeq Reveals Unbiased Cutting of the dHJ

In this study, we used RecSeq to examine the number, proportion and composition of final resolution signatures to better understand the relationship between Sgs1 and the ZMMs and their roles in regulating the CO-NCO decision. The recombination motifs we observed allowed us to attribute events arising from biased vs. unbiased cutting of the dHJ. Particularly, we have detected the appearance of apparent double COs (Type E5A) that are indicative of unbiased cleavage.

### A Model Incorporating the Roles of Sgs1, Zip3 and Msh4 in CO and NCO Formation

**Figure 6:**
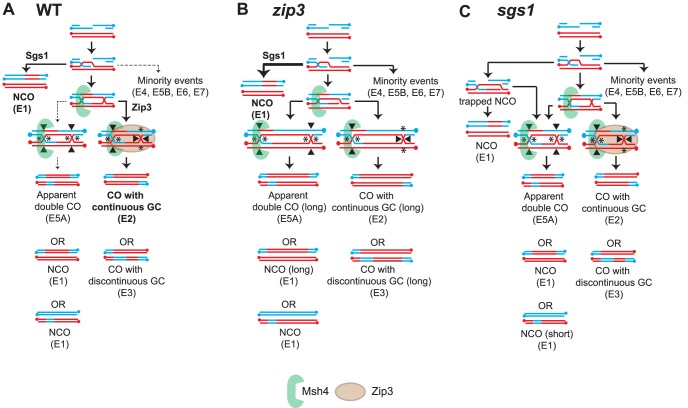
Model of the relationship between Sgs1, Zip3 and Msh4 in CO-NCO fate. Models for how (A) WT, (B) *zip3* and (C) *sgs1* might produce the distribution of observed recombination types. Indicated in bold type are the main signatures observed. Arrow weight indicates the relative degree of partitioning through the pathway. Expected biased or unbiased cuts are indicated by asterisks or filled arrowheads.

Figures 6A–6C depicts models for WT, *zip3* and *sgs1* respectively and how they can perturb the CO-NCO relationship to result in the observed recombination signatures and the changed proportion of COs and NCOs. Based on the results from the *zip3* mutant (Figure 6B), a key feature of the model for WT is that Zip3 is needed to ensure that biased resolution of the JM occurs. In *zip3*, E5A events increase substantially indicating that biased resolution has been perturbed or more likely completely lost given the 52% reduction of COs. In conjunction with unbiased cutting in *zip3*, an increase in the median length of both NCOs and GCco tracts is seen. Longer GCco tracts can arise if the two HJs making up the JM are farther apart increasing the heteroduplex length. If these HJs instead were cut in an unbiased manner, long NCOs would result thereby increasing the median NCO length as observed. Although Zip3 seems to play a role in directing biased cleavage, Zip3 also appears involved in stabilizing second end capture and promoting subsequent ligation to form the second HJ. This proposition stems from our observation that without Zip3, median GCco tracts are substantially longer. This extension is Sgs1-dependent since in *zip3 sgs1* GCco tracts are WT in length. We postulate from these findings that Zip3 limits the ability of Sgs1 to extend the D-loop and/or limits Sgs1-dependent resection, thus promoting ligation of the second HJ.

Another key feature in the model for wild-type CO-NCO balance is Sgs1's role in directing recombination both towards resolvase-independent NCO formation and towards ZMM-dependent CO formation as previously proposed by de Muyt et al. (2012). In *sgs1* (Figure 6C), the appearance of E5A events suggests that unbiased cleavage of dHJs is occurring in this mutant. An important function of Sgs1 is to disassociate the extended invading strand from the D-loop to promote the formation of NCOs; we speculate that in *sgs1* the lack of this ability will result in invasions originally destined to form NCOs becoming trapped in a JM-like intermediate (indistinguishable from JM on a 2D gel) that would require Cdc5 for release. In this trapped state, some strands will remain unligated but will presumably form NCOs upon Cdc5 induction by reannealing to the second end. In this case, a distinct population of small NCOs would form. We also speculate that a fraction of the trapped intermediate does become ligated, and is cut in an unbiased manner, giving rise to E5As. This model modifies a proposal by deMuyt et al. (2012) that suggests that in *sgs1*, NCOs mainly arise from JMs that are resolved by unbiased cutting. Our proposal of an unligated “trapped intermediate” was put forth in order to take into account the increase in the small NCO population (Figure S4). Unbiased cutting, although predicted to generate smaller NCOs, does not preferentially increase the frequency of small NCOs over NCOs of other sizes (Figure S1). Furthermore, *zip3 sgs1* shows no increase in the population of short NCOs even though there is an increase in E5A events furthering bolstering the argument unbiased cutting alone cannot explain the increase in short NCOs in *sgs1*.

### Msh4 Promotes Stability of the JM but Does Not Have a Role in Enforcing Biased Cutting

Unlike *zip3* and *sgs1*, the *msh4* mutant reveals no increase in E5As implying that biased designation is intact. In fact an *in vitro* study of hMSH4-hMSH5 [45] suggests that the role of Msh4-Msh5 is to recognize HJs and act as a meiosis-specific sliding clamp to stabilize dHJs. Our results showing that in *msh4* 1) GCco tracts are longer and 2) median GCco tract length remains long in *msh4 sgs1* is in agreement with such a proposed role. Because extension of the D-loop is dependent on Sgs1, we envisage that the GCco length increase seen in *msh4* is likely coming from the invading end of the DSB, which we speculate is not dependent on Sgs1 vs. the ligating end, which is dependent. If Msh4 normally stabilizes the invading end for JM formation, lack of this stabilization would cause the invading end to prematurely exit the D-loop and not get fully extended. Now this partially extended strand can invade again but this time the extended DNA is used as a primer for further extension, thus creating the potential for longer associated GCs. CO formation will be much more difficult to establish without sufficient JM stability thus accounting for the even lower amount of COs and spore viability than *zip3*. Note that NCO formation is not affected by loss of Msh4 since the number and lengths of NCOs are normal.

### The Subpopulation of Small NCOs in *sgs1* Is Potentially a Consequence of the Trapped NCO Intermediate

The trapped NCO intermediate postulated to form in *sgs1* contributes to the appearance of the short NCOs we see in *sgs1*. Normally during SDSA the invading strand repairs using the homolog as a template (Figure S4). After annealing the remaining resected region repairs using the reannealed strand. However, in *sgs1*, when the invading strand cannot release and becomes trapped in an unligated JM-like intermediate, the non-invading end can now repair using the homolog just as it would if there were a CO-intermediate. The presence of this additional small heteroduplex can result in a short NCO. In *sgs1 zip3*, no short NCOs are observed suggesting that Zip3 is needed to stabilize the trapped intermediate. This notion is bolstered by the fact that in *msh4 sgs1* in which Zip3 is present, a population of small NCOs is still discernable.

### Changes in Total Interhomolog Events May Reflect Changes in DSBs, Homolog Bias or Conversion

Recently, Thacker et al. (2014) showed that strains lacking ZMMs exhibit greater than wild-type levels of DSBs [46]. For the *zip3* mutant, this increase in DSBs is consistent with the increase in total interhomolog events (Table S1). However, in *msh4*, we find that total interhomolog events are decreased by 36%. One possibility is that more intersister repair than interhomolog repair occurs in *msh4*. However this seems unlikely since Oh et al. (2007) have shown that intersister repair decreases in *msh5*. We also cannot deduce if more NCOs were restored than converted. Although no decrease in the percentage of GC_co_s is observed (Table S1, E2% events with GC), any decrease would be obscured since GC_co_ conversion tracts are longer in *msh4* and thus increases detectability of GC_co_s. Therefore for this mutant, we cannot reliably deduce why total IH events decrease.

### Mms4 and Sgs1 Limit Multiple-Strand Invasions through Different Mechanisms

As previously reported, both *sgs1* and *mms4-md* showed an increase in the frequency of aberrant JMs as compared to WT [41]. However a difference was observed on how COs are now resolved in the different mutants [13]. In *sgs1*, a dependence on the Slx1-Slx4 resolvase was shown whereas in *mms4-md*, a role for the Yen1 resolvase was found. Our system for signature recognition also allowed us to detect important differences between the two mutants. In *sgs1*, multichromatid resolution patterns predominate whereas in *mms4-md*, there is a greater preponderance of multiple events on one or two chromatids. In Figure S5, we illustrate how mechanistically the multiple invasions might differ in the two mutants. In *sgs1*, multichromatid invasions are likely to arise from those events originally destined to be NCOs but trapped in JM-like intermediates. Because of the prolonged time in the trapped state, some of these will become ligated, forming JMs even without the presence of Zip3 and will resolve by unbiased cutting. The others are unligated. In this state, the 3′ end is still free to invade other chromatids increasing the probability of multichromatid events. On the other hand for *mms4-md*, multiple invasions arise when overextension of replication leads to a 3′ flap. Without the endonuclease activity of Mms4, ligation would be difficult thus permitting the 3′ end to invade again. This would manifest in both COs and NCOs. We suspect that the greater number of sequential invasions in *mms4-md* is the result of a combination of the low efficiency of ligation and the fact that Sgs1 is still around to divert the additional invasion into SDSA. Although both mutants will result in chromosome entanglements, the low efficiency of ligation might be a contributing factor why *mms4Δ* is more deleterious than *sgs1Δ* as evidenced by its poor spore viability.

## Materials and Methods

### Strain Construction

All yeast strains (see Text S1) were derived from a cross of haploid S96 and YJM789 parents. Most of the deletion strains were constructed by PCR mediated gene replacement using pFA6a-kanMX6 plasmid [47]. *mms4-md* strains were created by replacing the WT *MMS4* promoter with mitosis specific *CLB2* promoter using pFA6a-natMX4-pCLB2-3HA (pCA001) plasmid as a template. Haploid parents were mated for 8–12 hours before sporulation on 2% potassium-acetate plates at 30°C. Tetrads were dissected after 4–6 days. Colonies were streaked for single cells before growing up for DNA isolation.

Although 4-spore viable tetrads are used for the genomic analysis, the spore viability and sporulation frequencies of these strains are not at the level to trigger any major selection bias.

Actual bias has only been previously shown when the sporulation frequency was extremely poor (0.4%, 4-spore asci) as in the *zip1* mutant [39]. In fact, except in the case of very poor sporulation, the genomic analysis agrees quite well with published reports of recombination frequencies in the same mutants obtained by physical analyses using 1-D, 2-D gels and classical genetics [43]. It is also important to note that although we can obtain an average resolution of ∼80 bp, this analysis cannot detect 1) NCOs that been fully restored rather than converted; 2) events that solely involve sister chromatids and 3) any events that lie under the resolution defined by the density of SNPs (e.g. in conserved regions lacking SNPs).

### Sample Preparation

For most samples, genomic DNA was extracted using QIAGEN genomic tip 500/G from 100 mL overnight YPAD culture [48]. DNA library preparation was performed according to Illumina's protocol. Libraries from 4 or 16 spores were multiplexed using custom adapters (Text S1). Sequencing was performed at either Vincent J. Coates Genomics Sequencing Laboratory, UC Berkeley or at the Center for Advanced Technology, UCSF using Illumina's HiSeq sequencer. For four *msh4* tetrads (3,4,5 6), two *sgs1* tetrads (new1, new2) and *msh4sgs1x2*, sample preparation was performed using the NEXTflex DNA sequencing kit. For a list of barcodes used for multiplexing refer to Text S1.

### Data Analysis

Fastq files generated by Illumina's Casava pipeline were the starting point for all data analyses. ReCombine software package was used to perform alignment to reference genomes, genotype the markers and designate CO and NCO locations for each tetrad [31]. In order to categorize the complex recombination patterns seen in *mms4-md* and *sgs1* mutants, a custom analysis was performed using output data from ReCombine. To do so, the CrossOver program was run using a 0 kb range for close events instead of the default 5 kb. Next, COs and GCs within 5 kb of each other were grouped together as a single event. The categorization of events was performed based on the number of chromatids involved in the event and also the complexity of the event. Detailed segregation plots were generated using the plotSeg.R program [31]. Sequences will be available at SRA in BioProject #SRP028549 (WT) and #SRP041214 (mutants) and additional output from the analysis is deposited at Dryad Digital Depository (http://doi:10.5061/dryad.79hn1).

### Statistical Analyses

The t-test of means was used to calculate statistical significance in the average number of events between mutants. To compare tract lengths, the non-parametric Wilcox test was used to compute statistical significance. Comparison between proportions was performed using the z test of proportions. A Kolmogorov-Smirnov test was used to calculate the statistical significance between different tract length distributions. P-values were not corrected for multiple comparisons.

## Supporting Information

Figure S1 Possible products of dHJ resolution. (A) Biased resolution of a dHJ can generate an E2 when the repaired strand gets cleaved at the second end capture end (purple cuts). If the strands are cleaved in the other orientation (green cuts), the expected products are E2, E3 and E5. (B) Unbiased resolution of dHJ can lead to E5A or E1 events. In both cases, depending how the heteroduplex intermediate is repaired generates different products.
(EPS)

Figure S2 *sgs1* shows increased number of minority events specifically involving complex events with multiple chromatids and tracts. (A) Multi-chromatid JMs arising from a single DSB can give rise to E5, E6 and E7 events in *sgs1*. Events involving two, three or four chromatids and more than one dHJ can result in multi-chromatid resolution patterns. (B) Classification of CO events by number of strands and complexity. *sgs1* shows a decrease in the proportion of simple COs and an increase in the proportion of apparent double COs and more complex CO involving either two, three or four strands. Error bars represent standard error of proportions. (C) Proportions of events plotted as percent of total and classified by number of chromatids involved for WT and *sgs1*. Error bars indicate standard error of proportions.
(EPS)

Figure S3 E5A events representing unbiased resolution of JMs make up for the majority of E5 events. (A) E5 events can be classified into: E5A – signature representing unbiased resolution of JM, E5B_1_ – Two NCOs within 5 kb of each other on a pair of homologous chromatids, E5B_2_ – Two NCOs within 5 kb of each other on a pair of sister chromatids, E5B_3_ – all other complex events including triple COs. The numbers in parenthesis are total number of each type observed in wild-type data. (B) Plot representing the total number of E5 event types in wild-type data. Type E5A form the majority of all E5 events.
(EPS)

Figure S4 Trapped SDSA intermediate can form short NCOs in absence of Sgs1. In (A) WT, SDSA forms a single heteroduplex resulting from the repair of the invading strand using the homolog. In (B) *sgs1*, a trapped JM-like intermediate can form that could allow the non-invading resected end also to be repaired by the homolog. The resulting short heteroduplex could form a short NCO.
(EPS)

Figure S5 Multi chromatid JMs in *sgs1* and *mms4-md* form by different mechanisms. (A) In *mms4-md*, when the 3′ flap remains unrepaired it can invade again. However, the second invasion is more likely to be disassembled by Sgs1 and thus repaired via SDSA. This would result in the repair visible on the invading strand alone creating discontinuities. (B) In *sgs1*, NCOs trapped in JM-like intermediates can participate in additional invasions. Since Sgs1 is absent, these intermediates form ligated JMs and get resolved by dHJ resolution pathway giving rise to resolution patterns involving multiple chromatids.
(EPS)

Table S1 Comparison of number of events, tract lengths (bp), spore viability and spore efficiency between the analyzed set of mutants.
(DOCX)

Table S2 p values obtained using t-test to compare average number of events per tetrad.
(DOCX)

Table S3 p values obtained using Wilcox test to compare tract lengths.
(DOCX)

Text S1 Supplemental experimental procedures containing a list of strains and a list of sequenced samples and accompanying bar codes.
(PDF)
