# Correction: Controlling Meiotic Recombinational Repair – Specifying the Roles of ZMMs, Sgs1 and Mus81/Mms4 in Crossover Formation

**DOI:** 10.1371/journal.pgen.1004870

**Published:** 2014-11-12

**Authors:** The PLOS Genetics Staff

In the Data Availability section, the URL for accessing the data deposited at Dryad Digital Depository is incorrect. The correct URL is: http://doi.org/10.5061/dryad.79hn1.
