# Supplementary material for: Controlling Meiotic Recombinational Repair – Specifying the Roles of ZMMs, Sgs1 and Mus81/Mms4 in Crossover Formation
Source: PLoS Genet. 2014 Oct 16;10(10):e1004690. doi: 10.1371/journal.pgen.1004690 (PMC4199502; doi:10.1371/journal.pgen.1004690)
Supplement: Table S1 — Comparison of number of events, tract lengths (bp), spore viability and spore efficiency between the analyzed set of mutants. (DOCX) [file pgen.1004690.s006.docx]

|  | wild type | *sgs1* | *zip3* | *zip3*  *sgs1* | *msh4* | *msh4*  *sgs1* | *mms4-md* | *msh2* | *mms4-md*  *msh2* |
| --- | --- | --- | --- | --- | --- | --- | --- | --- | --- |
| Number of tetrads | 52 | 11 | 7 | 4 | 7 | 5 | 7 | 4 | 4 |
| E1 *Single NCO* total# | 1844 | 596 | 722 | 230 | 351 | 233 | 363 | 161 | 216 |
| E1 Average# per tetrad | 35.5 | 54.2 | 103.1 | 57.5 | 50.1 | 46.6 | 51.9 | 40.2 | 54 |
| E1 event length Median | 1777.8 | 1541.2 | 1958.8 | 1678.2 | 1679 | 2014 | 2734 | 945 | 813 |
| E1 event length Mean | 2152.9 | 2178 | 2520.4 | 2156.1 | 2028.1 | 2550.3 | 3844 | 1208.1 | 2374.5 |
| E2 *Single CO* total# | 4008 | 812 | 272 | 236 | 200 | 309 | 454 | 322 | 260 |
| E2 Average# per tetrad | 77.7 | 73.8 | 38.9 | 59 | 28.6 | 61.8 | 64.9 | 80.5 | 65 |
| E2 GC length Median | 1917 | 2072 | 3488 | 1987.5 | 2835 | 2731 | 3334.5 | 911.8 | 939.2 |
| E2 GC length Mean | 2321.9 | 2547.4 | 4167.1 | 2430.8 | 3298.8 | 3044.5 | 4467.3 | 1254.2 | 2423.2 |
| E2 % events with GC | 64.8 | 65.4 | 89 | 70.8 | 83 | 63.1 | 73.8 | 51.6 | 51.5 |
| E3 *Discontinuous GC* total# | 437 | 168 | 60 | 52 | 24 | 72 | 142 | 92 | 84 |
| E3 Average# per tetrad | 8.4 | 15.3 | 8.6 | 13 | 3.4 | 14.4 | 20.3 | 23 | 21 |
| E3 event length Median | 2929 | 3364.5 | 4437 | 2911.5 | 4276.5 | 3336.5 | 5249.5 | 1513.5 | 2877.5 |
| E3 event length Mean | 3139.4 | 3587.6 | 4889 | 3418.1 | 4320.5 | 3689.9 | 5524 | 1910.1 | 3799.3 |
| E3 GC length Median | 829 | 1211.5 | 2620.5 | 1571.8 | 2625.2 | 1684.5 | 1908.5 | 555 | 596.5 |
| E3 GC length Mean | 1212.5 | 1768.5 | 2975.1 | 1721.9 | 2555.6 | 2085.2 | 2556.1 | 749.2 | 1378.8 |
| E3 NCO length Median | 996.2 | 868.8 | 615 | 864.8 | 990 | 939 | 776 | 390.2 | 505.5 |
| E3 NCO length Mean | 1199.8 | 1407.3 | 1070.7 | 1006.9 | 1345.4 | 1431.2 | 1299.8 | 548.7 | 896.9 |
| E4 *Discontinuous NCO* total# | 47 | 41 | 26 | 9 | 6 | 11 | 89 | 18 | 41 |
| E4 Average# per tetrad | 0.9 | 3.7 | 3.7 | 2.2 | 0.9 | 2.2 | 12.7 | 4.5 | 10.2 |
| E4 event length Median | 3329 | 3690 | 3728 | 3348 | 4693.5 | 4242 | 5345 | 1577.5 | 3241 |
| E4 event length Mean | 3789 | 3809 | 4218.9 | 4132.7 | 4882.7 | 4553 | 6089.7 | 1906.6 | 4284.5 |
| E4 tract length Median | 790 | 978 | 558.2 | 940 | 509.2 | 1203.5 | 893 | 462 | 509.5 |
| E4 tract length Mean | 1269 | 1392.9 | 1551.2 | 1397.3 | 1427.9 | 1626.7 | 1585.4 | 730.4 | 1144.7 |
| E5 *Minority on two* total# | 165 | 157 | 85 | 52 | 25 | 72 | 94 | 31 | 38 |
| E5 Average# per tetrad | 3.2 | 14.3 | 12.1 | 13 | 3.6 | 14.4 | 13.4 | 7.8 | 9.5 |
| E5 event length Median | 3455 | 3845 | 4693.5 | 3129 | 4220 | 3473.5 | 5532 | 2455 | 3846.5 |
| E5 event length Mean | 3681.4 | 4273.7 | 5158.2 | 3917.7 | 4877.1 | 3801.1 | 6855.2 | 2908.8 | 5538.8 |
| E5A *Apparent double CO on two* total# | 82 | 63 | 25 | 24 | 4 | 32 | NA | NA | NA |
| E5A Average# per tetrad | 1.6 | 5.7 | 3.6 | 6 | 0.6 | 6.4 | NA | NA | NA |
| E6 *Minority on three* total# | 266 | 148 | 66 | 48 | 20 | 81 | 74 | 28 | 24 |
| E6 Average# per tetrad | 5.1 | 13.5 | 9.4 | 12 | 2.9 | 16.2 | 10.6 | 7 | 6 |
| E6 event length Median | 3461 | 3763.5 | 5594 | 3569 | 5307.5 | 4454 | 5508 | 3157.5 | 5209.5 |
| E6 event length Mean | 3752.6 | 4254.4 | 5871.4 | 4031.9 | 5453.8 | 5299.5 | 7023.4 | 3855.2 | 6488.7 |
| E6 GC length Median | 1273.2 | 1294.8 | 1105 | 1657 | 2212 | 1142.8 | 1456.2 | 814 | 1072 |
| E6 GC length Mean | 1503.1 | 1713.5 | 1844 | 1810.2 | 2278.5 | 1690.3 | 2069.6 | 914.2 | 2124.2 |
| E6 NCO length Median | 1485 | 790 | 803 | 1198.5 | 1097 | 1039.5 | 597 | 631.8 | 820 |
| E6 NCO length Mean | 1901.7 | 1313.1 | 1271.6 | 1791.5 | 1903.9 | 1621.5 | 1542.1 | 869.6 | 1373.9 |
| E7 *Minority on four* total# | 36 | 34 | 6 | 9 | 3 | 12 | 8 | 4 | 3 |
| E7 Average# per tetrad | 0.7 | 3.1 | 0.9 | 2.2 | 0.4 | 2.4 | 1.1 | 1 | 0.8 |
| E7 event length Median | 4298.5 | 4468.5 | 4997.5 | 3771 | 2830 | 6472 | 8110 | 1360.5 | 11652 |
| E7 event length Mean | 4405.6 | 5076.4 | 6307.2 | 4858.9 | 2500 | 6086.8 | 7978.9 | 1766.8 | 11767.3 |
| E7 GC length Median | 1093.8 | 1725.5 | 1986 | 1822.8 | NA | 1785 | 2290.5 | 773.5 | 1863.2 |
| E7 GC length Mean | 1247.6 | 2018 | 1986 | 1689.2 | NA | 2310.9 | 2376 | 773.5 | 1926.8 |
| E7 NCO length Median | 1986 | 906.5 | 2836.5 | 555.8 | NA | 515.8 | 2160.8 | 72.2 | 565.5 |
| E7 NCO length Mean | 2144.4 | 1341.1 | 3044.5 | 1040.3 | NA | 953.3 | 2404.7 | 72.2 | 1395.8 |
| Total single dHJ CO# (E2,E3) | 4445 | 980 | 332 | 288 | 224 | 381 | 596 | 414 | 344 |
| Average total single dHJ CO# per tetrad | 85.5 | 89.1 | 47.4 | 72 | 32 | 76.2 | 85.1 | 103.5 | 86 |
| Total JM#(E2,E3,E5,E6,E7) | 4912 | 1319 | 489 | 397 | 272 | 546 | 772 | 477 | 409 |
| Average Total JM# per tetrad | 94.5 | 119.9 | 69.9 | 99.2 | 38.9 | 109.2 | 110.3 | 119.2 | 102.2 |
| Disc NCO Gap length Median | 721 | 588.5 | 384.5 | 764.8 | 612.5 | 399.5 | 429.5 | 340.8 | 609.5 |
| Disc NCO Gap length Mean | 948.8 | 824.9 | 570 | 792 | 867.3 | 620.2 | 772.7 | 401 | 896.6 |
| Disc GC Gap length Median | 1242.2 | 1013.5 | 930 | 1327 | 1257 | 1121.5 | 1175 | 733.2 | 791 |
| Disc GC Gap length Mean | 1524.4 | 1409.8 | 1838.2 | 1648 | 1684.3 | 1526.2 | 1735 | 930.8 | 1207.7 |
| Total NCO# (E1+E4) | 1891 | 637 | 748 | 239 | 357 | 244 | 452 | 179 | 257 |
| Average total NCO# per tetrad | 36.4 | 57.9 | 106.9 | 59.8 | 51 | 48.8 | 64.6 | 44.8 | 64.2 |
| Total IH events # | 6803 | 1956 | 1237 | 636 | 629 | 790 | 1212 | 652 | 658 |
| Average Total IH# per tetrad | 130.8 | 177.8 | 176.7 | 159 | 89.9 | 158 | 173.1 | 163 | 164.5 |
| Spore Viability (% viable) | 86.1 | 62.3 | 62.7 | 51.1 | 33.6 | 52.3 | 25.9 | 69.4 | 36.4 |
| # tetrads used to score viability | 209 | 203 | 211 | 251 | 245 | 208 | 1165 | 121 | 381 |
| Sporulation Efficiency (%, total) | 77 (300) | 45 (301) | 62 (614) | 12 (298) | 33 (656) | 35 (300) | 29 (601) | 65 (604) | 35 (701) |
