# Supplementary material for: Controlling Meiotic Recombinational Repair – Specifying the Roles of ZMMs, Sgs1 and Mus81/Mms4 in Crossover Formation
Source: PLoS Genet. 2014 Oct 16;10(10):e1004690. doi: 10.1371/journal.pgen.1004690 (PMC4199502; doi:10.1371/journal.pgen.1004690)
Supplement: Table S2 — p values obtained using t-test to compare average number of events per tetrad. (DOCX) [file pgen.1004690.s007.docx]

| genotype pair | | E1 | E2 | E3 | E4 | E5 | E6 | E7 | Total NCO | Total Simple CO | Total JM | Total IH |
| --- | --- | --- | --- | --- | --- | --- | --- | --- | --- | --- | --- | --- |
| wild type | *sgs1* | <0.001 | 0.32 | <0.001 | <0.001 | <0.001 | <0.001 | <0.001 | <0.001 | 0.21 | <0.001 | <0.001 |
| wild type | *zip3* | <0.001 | <0.001 | 0.91 | 0.02 | <0.001 | 0.08 | 0.58 | <0.001 | <0.001 | <0.001 | 0.01 |
| wild type | *zip3 sgs1* | 0.01 | 0.1 | 0.31 | 0.06 | <0.001 | 0.03 | 0.04 | 0.01 | 0.06 | 0.63 | 0.01 |
| wild type | *msh4* | <0.001 | <0.001 | <0.001 | 0.88 | 0.68 | <0.001 | 0.43 | <0.001 | <0.001 | <0.001 | <0.001 |
| wild type | *msh4 sgs1* | 0.06 | 0.04 | 0.02 | 0.15 | <0.001 | 0.01 | <0.001 | 0.06 | 0.09 | 0.09 | 0.07 |
| *sgs1* | *zip3* | <0.001 | <0.001 | <0.001 | 0.99 | 0.39 | 0.03 | <0.001 | <0.001 | <0.001 | <0.001 | 0.9 |
| *sgs1* | *zip3 sgs1* | 0.55 | 0.15 | 0.6 | 0.08 | 0.5 | 0.18 | 0.24 | 0.74 | 0.03 | 0.02 | 0.03 |
| *sgs1* | *msh4* | 0.35 | <0.001 | <0.001 | <0.001 | <0.001 | <0.001 | <0.001 | 0.13 | <0.001 | <0.001 | <0.001 |
| *sgs1* | *msh4 sgs1* | 0.22 | 0.08 | 0.68 | 0.14 | 0.95 | 0.36 | 0.22 | 0.17 | 0.03 | 0.19 | 0.16 |
| *zip3* | *zip3 sgs1* | <0.001 | 0.07 | 0.33 | 0.19 | 0.73 | 0.37 | 0.05 | <0.001 | 0.01 | 0.01 | 0.2 |
| *zip3* | *msh4* | <0.001 | 0.02 | 0.01 | 0.02 | <0.001 | 0.01 | 0.3 | <0.001 | 0.01 | <0.001 | <0.001 |
| *zip3* | *msh4 sgs1* | <0.001 | 0.01 | 0.03 | 0.23 | 0.41 | 0.04 | <0.001 | <0.001 | <0.001 | <0.001 | 0.29 |
| *zip3 sgs1* | *msh4* | 0.16 | 0.03 | 0.08 | 0.05 | <0.001 | 0.01 | 0.02 | 0.11 | <0.001 | <0.001 | <0.001 |
| *zip3 sgs1* | *msh4 sgs1* | 0.1 | 0.77 | 0.75 | 0.96 | 0.53 | 0.11 | 0.79 | 0.12 | 0.53 | 0.23 | 0.99 |
| *msh4* | *msh4 sgs1* | 0.49 | <0.001 | <0.001 | 0.15 | <0.001 | <0.001 | <0.001 | 0.68 | <0.001 | <0.001 | <0.001 |
| wild type | *mms4-md* | <0.001 | 0.04 | 0.01 | <0.001 | <0.001 | <0.001 | 0.32 | <0.001 | 0.94 | 0.01 | <0.001 |
| wild type | *msh2* | 0.34 | 0.63 | 0.02 | 0.13 | 0.04 | 0.54 | 0.64 | 0.25 | 0.02 | 0.01 | 0.02 |
| wild type | *mms4*  *msh2* | <0.001 | 0.01 | 0.03 | <0.001 | 0.02 | 0.36 | 0.84 | <0.001 | 0.92 | 0.46 | 0.02 |
| *mms4-md* | *msh2* | 0.07 | 0.09 | 0.55 | 0.02 | 0.03 | 0.1 | 0.85 | 0.03 | 0.02 | 0.14 | 0.27 |
| *mms4-md* | *mms4*  *msh2* | 0.62 | 0.98 | 0.87 | 0.35 | 0.12 | 0.01 | 0.43 | 0.95 | 0.9 | 0.34 | 0.37 |
| *msh2* | *mms4*  *msh2* | 0.04 | 0.09 | 0.69 | 0.04 | 0.43 | 0.25 | 0.71 | 0.04 | 0.03 | 0.07 | 0.89 |
