# Supplementary material for: Controlling Meiotic Recombinational Repair – Specifying the Roles of ZMMs, Sgs1 and Mus81/Mms4 in Crossover Formation
Source: PLoS Genet. 2014 Oct 16;10(10):e1004690. doi: 10.1371/journal.pgen.1004690 (PMC4199502; doi:10.1371/journal.pgen.1004690)
Supplement: Table S3 — p values obtained using Wilcox test to compare tract lengths. (DOCX) [file pgen.1004690.s008.docx]

| genotype pair | | GC_CO_ | E1 | E4 | E4  tract | E4  gap |
| --- | --- | --- | --- | --- | --- | --- |
| wild type | *sgs1* | 0.057 | 0.001 | NC | NC | NC |
| wild type | *zip3* | <0.001 | <0.001 | NC | NC | NC |
| wild type | *zip3sgs1* | 0.36 | 0.52 | NC | NC | NC |
| wild type | *msh4* | <0.001 | 0.19 | NC | NC | NC |
| wild type | *msh4sgs1* | <0.001 | 0.21 | NC | NC | NC |
| *sgs1* | *zip3* | <0.001 | <0.001 | NC | NC | NC |
| *sgs1* | *zip3sgs1* | 0.76 | 0.09 | NC | NC | NC |
| *sgs1* | *msh4* | <0.001 | 0.13 | NC | NC | NC |
| *sgs1* | *msh4sgs1* | <0.001 | 0.004 | NC | NC | NC |
| *zip3* | *zip3sgs1* | <0.001 | 0.01 | NC | NC | NC |
| *zip3* | *msh4* | 0.003 | <0.001 | NC | NC | NC |
| *zip3* | *msh4sgs1* | <0.001 | 0.42 | NC | NC | NC |
| *zip3sgs1* | *msh4* | <0.001 | 0.76 | NC | NC | NC |
| *zip3sgs1* | *msh4sgs1* | 0.001 | 0.22 | NC | NC | NC |
| *msh4* | *msh4sgs1* | 0.23 | 0.28 | NC | NC | NC |
| wild type | *mms4-md* | <0.001 | <0.001 | <0.001 | 0.83 | 0.04 |
| wild type | *msh2* | <0.001 | <0.001 | <0.001 | 0.02 | <0.001 |
| wild type | *mms4-md msh2* | <0.001 | <0.001 | 0.78 | 0.04 | 0.42 |
| *mms4-md* | *msh2* | <0.001 | <0.001 | <0.001 | 0.02 | 0.16 |
| *mms4-md* | *mms4-md msh2* | <0.001 | <0.001 | 0.01 | 0.02 | 0.22 |
| *msh2* | *mms4-md msh2* | 0.13 | 0.37 | <0.001 | 0.58 | 0.03 |
