# Supplementary material for: Controlling Meiotic Recombinational Repair – Specifying the Roles of ZMMs, Sgs1 and Mus81/Mms4 in Crossover Formation
Source: PLoS Genet. 2014 Oct 16;10(10):e1004690. doi: 10.1371/journal.pgen.1004690 (PMC4199502; doi:10.1371/journal.pgen.1004690)
Supplement: Text S1 — Supplemental experimental procedures containing a list of strains and a list of sequenced samples and accompanying bar codes. (PDF) [file pgen.1004690.s009.pdf]

List of strains

Strain	Genotype
S96	*MATa ho lys5*
YJM789	*MATα ho::hisG lys2 cyh*
SYC1120	S96 but *sgs1Δ::KanMX6*
SYC1121	YJM789 but *sgs1Δ::KanMX6*
YCA007	S96 but *Clb2 mms4::NatMX6*
YCA008	YJM789 but *Clb2 mms4::NatMX6*
YPY166	S96 but *msh2Δ::KanMX6*
YPY168	YJM789 but *msh2Δ::KanMX6*
YPY79, YPY80	S96 but *Clb2mms4::NatMX6 msh2::KanMX6*
YPY82, YPY83	YJM789 but *Clb2mms4::NatMX6 msh2::KanMX6*
SYC1110	S96 but *msh4Δ::KanMX6*
SYC1111	YJM789 but *msh4Δ::KanMX6*
YCA1078, YCA 1088	S96 but *msh4Δ::KanMX6 sgs1Δ::NAT*
YCA1079, YCA 1081	YJM789 but *msh4Δ::KanMX6 sgs1Δ::NAT*
YCA1130, YCA1131	S96 but *zip3Δ::KanMX6 sgs1Δ::NAT*
YCA1083, YCA1084	YJM789 but *zip3Δ::KanMX6 sgs1Δ::NAT*
YCA1318, YCA1319, YCA1320	S96 but *sgs1Δ::NAT*
YCA1321, YCA1322, YCA1323	YJM789 *sgs1Δ::NAT*
YCA223, YCA224, YCA225	S96 but *zip3Δ::KanMX6*
YCA291, YCA292, YCA293	YJM789 *zip3Δ::KanMX6*

List of sequenced samples

Tetrad name	Sample Prep	Sequencing Platform	Spore	Barcode
wtx29	Illumina Protocol	Illumina Genome Analyzer II	a	TGT
			b	GTT
			c	CAT
			d	ACT
wtx30	Illumina Protocol	Illumina Genome Analyzer II	a	-
			b	-
			c	-
			d	-
wtx46	Illumina Protocol	Illumina Genome Analyzer II	a	TGT
			b	GTT
			c	CAT
			d	ACT
wtx63	Illumina protocol	Illumina Genome Analyzer II	a	TGT
			b	GTT
			c	CAT
			d	ACT
wtx64	Illumina protocol	Illumina Genome Analyzer II	a	TGT
			b	GTT
			c	CAT
			d	ACT
wtx65	Illumina protocol	Illumina Genome Analyzer II	a	TGT
			b	GTT
			c	CAT
			d	ACT
sgs1x2	Illumina protocol	Illumina Genome Analyzer II	a	TGT
			b	GTT
			c	CAT
			d	ACT
sgs1x7	Illumina protocol	Illumina Genome Analyzer II	a	TGT
			b	GTT
			c	CAT
			d	ACT
sgs1x8	Illumina protocol	Illumina Genome Analyzer II	a	TGT
			b	GTT
			c	CAT
			d	ACT
sgs1x30	Illumina protocol	HiSeq 2000	a	TGACT
			b	GTCAT

			c	CAGTT
			d	ACTGT
sgs1x32	Illumina protocol	HiSeq 2000	a	TGGTT
			b	GTTGT
			c	CAACT
			d	ACCTT
sgs1x33	Illumina protocol	HiSeq 2000	a	TGCAT
			b	GTACT
			c	CATGT
			d	ACGTT
sgs1x34	Illumina protocol	HiSeq 2000	a	TGTGT
			b	GTGTT
			c	CACAT
			d	ACACT
sgs1x35	Illumina protocol	HiSeq 2000	a	TGTGT
			b	GTGTT
			c	CACAT
			d	ACACT
sgs1x36	Illumina protocol	HiSeq 2000	a	TGGTT
			b	GTTGT
			c	CAACT
			d	ACCAT
sgs1newx1	NextFlex kit	HiSeq 2000	a	NextFlex#17
			b	NextFlex#18
			c	NextFlex#19
			d	NextFlex#20
sgs1newx2	NextFlex kit	HiSeq 2000	a	NextFlex#21
			b	NextFlex#22
			c	NextFlex#23
			d	NextFlex#24
msh4x1	Illumina Protocol	Illumina Genome Analyzer II	a	TGT
			b	GTT
			c	CAT
			d	ACT
msh4x2	Illumina Protocol	Illumina Genome Analyzer II	a	TGT
			b	GTT
			c	CAT
			d	ACT
msh4x8	Illumina Protocol	Illumina Genome Analyzer II	a	-
			b	-
			c	-
			d	-

msh4x3	NextFlex kit	HiSeq 2000	a	NextFlex#17
			b	NextFlex#18
			c	NextFlex#19
			d	NextFlex#20
msh4x4	NextFlex kit	HiSeq 2000	a	NextFlex#21
			b	NextFlex#22
			c	NextFlex#23
			d	NextFlex#24
msh4x5	NextFlex kit	HiSeq 2000	a	NextFlex#25
			b	NextFlex#26
			c	NextFlex#27
			d	NextFlex#28
msh4x6	NextFlex kit	HiSeq 2000	a	NextFlex#29
			b	NextFlex#30
			c	NextFlex#31
			d	NextFlex#32
msh4sgs1x2	NextFlex kit	HiSeq 2000	a	NextFlex#17
			b	NextFlex#18
			c	NextFlex#19
			d	NextFlex#20
msh4sgs1x3	NextFlex kit	HiSeq 2000	a	NextFlex#25
			b	NextFlex#26
			c	NextFlex#27
			d	NextFlex#28
msh4sgs1x6	NextFlex kit	HiSeq 2000	a	NextFlex#21
			b	NextFlex#22
			c	NextFlex#23
			d	NextFlex#24
msh4sgs1x8	NextFlex kit	HiSeq 2000	a	NextFlex#25
			b	NextFlex#26
			c	NextFlex#27
			d	NextFlex#28
msh4sgs1x10	NextFlex kit	HiSeq 2000	a	NextFlex#29
			b	NextFlex#30
			c	NextFlex#31
			d	NextFlex#32
zip3x262	Illumina Protocol	Illumina Genome Analyzer II	a	TGT
			b	GTT
			c	CAT
			d	ACT
zip3x265	Illumina protocol	HiSeq 2000	a	TGCAT
			b	GTACT

			c	CATGT
			d	ACGTT
zip3x268	Illumina Protocol	Illumina Genome Analyzer II	a	TGT
			b	GTT
			c	CAT
			d	ACT
zip3x274	Illumina protocol	HiSeq 2000	a	TGACT
			b	GTCAT
			c	CAGTT
			d	ACTGT
zip3x276	Illumina protocol	HiSeq 2000	a	TGTGT
			b	GTGTT
			c	CACAT
			d	ACACT
zip3x289	Illumina protocol	HiSeq 2000	a	TGGTT
			b	GTTGT
			c	CAACT
			d	ACCAT
zip3x295	Illumina Protocol	Illumina Genome Analyzer II	a	TGT
			b	GTT
			c	CAT
			d	ACT
zip3sgs1x4	NextFlex kit	HiSeq 2000	a	NextFlex#1
			b	NextFlex#2
			c	NextFlex#3
			d	NextFlex#4
zip3sgs1x5	NextFlex kit	HiSeq 2000	a	NextFlex#5
			b	NextFlex#6
			c	NextFlex#7
			d	NextFlex#8
zip3sgs1x8	NextFlex kit	HiSeq 2000	a	NextFlex#9
			b	NextFlex#10
			c	NextFlex#11
			d	NextFlex#12
zip3sgs1x10	NextFlex kit	HiSeq 2000	a	NextFlex#13
			b	NextFlex#14
			c	NextFlex#15
			d	NextFlex#16
mms4x1	Illumina Protocol	Illumina Genome Analyzer II	a	TGT
			b	GTT
			c	CAT
			d	ACT

mms4x2	Illumina Protocol	Illumina Genome Analyzer II	a	TGT
			b	GTT
			c	CAT
			d	ACT
mms4x4	Illumina Protocol	Illumina Genome Analyzer II	a	TGT
			b	GTT
			c	CAT
			d	ACT
mms4x21	Illumina protocol	HiSeq 2000	a	TGGCT
			b	GTATT
			c	CATAT
			d	ACCGT
mms4x22	Illumina protocol	HiSeq 2000	a	TAGAT
			b	GCAGT
			c	CGTCT
			d	ATCTT
mms4x23	Illumina protocol	HiSeq 2000	a	TTGGT
			b	GGAAT
			c	CCTTT
			d	AACCT
mms4x24	Illumina protocol	HiSeq 2000	a	TCGTT
			b	GAACT
			c	CTTGT
			d	AGCAT
msh2x1	Illumina protocol	HiSeq 2000	a	TGGCT
			b	GTATT
			c	CATAT
			d	ACCGT
msh2x3	Illumina protocol	HiSeq 2000	a	TAGAT
			b	GCAGT
			c	CGTCT
			d	ATCTT
msh2x4	Illumina protocol	HiSeq 2000	a	TTGGT
			b	GGAAT
			c	CCTTT
			d	AACCT
msh2x5	Illumina protocol	HiSeq 2000	a	TCGTT
			b	GAACT
			c	CTTGT
			d	AGCAT
mms4msh2x1	Illumina protocol	HiSeq 2000	a	TATT
			b	GGCT

			c	CCGT
			d	ATAT
mms4msh2x2	Illumina protocol	HiSeq 2000	a	TCTT
			b	GTCT
			c	CAGT
			d	AGAT
mms4msh2x3	Illumina protocol	HiSeq 2000	a	TGGT
			b	GAAT
			c	CTTT
			d	ACCT
mms4msh2x5	Illumina protocol	HiSeq 2000	a	TTGT
			b	GCAT
			c	CGTT
			d	AACT
